# Women’s compliance with cervical cancer screening recommendations and associated barriers to participation: A cross-sectional analysis

**DOI:** 10.1371/journal.pone.0357819

**Published:** 2026-09-10

**Authors:** Lauma Spriņģe, Anda Ķīvīte-Urtāne, Ļubova Tihomirova, Inese Stars, Elza Mikule, Jana Žodžika, Aija Bukova-Žideļūna, Kersti Pärna, Mari Nygard, Anneli Uusküla

**Affiliations:** 1 Institute of Public Health, Riga Stradiņš University, Riga, Latvia; 2 Gynaecology Department, Riga East Clinical University Hospital, Riga, Latvia; 3 Institute of Family Medicine and Public Health, University of Tartu, Tartu, Estonia; 4 Cancer Registry of Norway, Norwegian Institute of Public Health, Oslo, Norway; University of Arkansas for Medical Sciences, UNITED STATES OF AMERICA

## Abstract

Cervical cancer screening (CCS) programmes play a key role in the early detection of cervical cancer (CC) and in reducing related morbidity and mortality. However, both under and overscreening deviate from evidence-based guidelines and can result in more harm rather than benefit. This cross-sectional study is aimed to evaluate the compliance of the CCS target group with the national CCS programme and to identify factors associated with different screening frequencies, including sociodemographic characteristics, health status and behaviour, awareness of the prevention of CC and the venue of the study participants’ recruitment. A cross-sectional survey was conducted among 1,279 women aged 25–70 years residing in Latvia. Multinomial logistic regression was used to assess associations between CCS frequency (once every three years, biannually or more often, once every four years or less, and “do not remember”) and independent variables, such as self-assessed health, chronic diseases, smoking and binge drinking, condom use and number of sexual partners and awareness of CCS invitation frequency, understanding of the frequency and purpose of the screening test, and knowledge of HPV vaccination, as well as venue of recruitment of study participants (colposcopy patients or general population). All variables were adjusted for key sociodemographic covariates. Overscreening (biannually or more often), underscreening (once every four years or less), and not recalling CCS history were strongly associated with a lack of awareness about the national CCS guidelines (e.g., unawareness that CCS is offered every three years). Overscreening was more common among participants recruited from colposcopy clinic. The results underscored the importance of health literacy in promoting appropriate screening behaviours. Addressing knowledge gaps through accessible and culturally relevant educational interventions could advance equity and enhance the effectiveness of CC prevention strategies.

## Introduction

Cervical cancer (CC) remains one of the leading causes of cancer-related mortality among women worldwide [1]. In Latvia, CC is the second leading cause of cancer‑related death among women aged 15–44 years, with a mortality rate of 4.2 per 100,000 and an incidence rate of 23.9 per 100,000, ranking the country third in the EU for the numbers associated or diagnosed with the disease [2]. Organised cervical cancer screening (CCS) programmes reduce overall CC mortality. A systematic review reported a 41–87% reduction in CC mortality among women participating in CCS in Northern Europe [3]. Another study estimated that the introduction prevented up to 49% of expected cases of CC over a 50-year period [4]. Hence, despite considerable prevention efforts, substantial differences in CC incidence and mortality persist even in countries with established CCS programmes [5], highlighting the importance of further investigation to better understand the factors that influence CCS effectiveness.

Latvia introduced organised CCS in 2009, targeting women aged 25–70 years. The National Health Service (NHS) sends individual invitation letters to eligible women at three‑year intervals, directing them to schedule appointments with contracted clinics. Initially, CCS was based on cytological testing; however, since July 2022, HPV testing has been introduced every five years for women aged 30–70 years, while cytology remains the primary test for women aged 25–29 [6]. CCS can also occur opportunistically during routine gynaecology visits. Between 2016 and 2018, 65% of women underwent CCS, with more than half, particularly younger women, opting for testing outside the organised programme [7]. NHS data from 2023 reported a 55.2% participation rate based on registered healthcare procedures [8], while national survey data from 2022 showed that 68.6% of women self-reported CCS uptake within the past three years. Both figures fall short of the 85% participation rate recommended by European guidelines [9,10]. Moreover, national statistics do not capture CCS procedures performed out-of-pocket in private clinics.

Most research on CCS participation has traditionally focused on overall uptake and barriers to attendance (i.e., reasons for non-participation). However, such an approach can mask two underexplored but increasingly important patterns: overscreening (screening more frequently than recommended) and screening history uncertainty (inability to recall screening frequency or timing) [11–13]. Both patterns matter. Overscreening is a form of low-value care [14] that can expose women to unnecessary follow-up procedures and psychosocial harms [15–17] while increasing system costs [18], while the inability to recall screening history may reflect limited engagement, inadequate health literacy, and weak integration of screening into routine preventive behaviours that can contribute to delay in diagnosis and unfavourable outcomes [19,20] and contribute to higher CC rates [11,21].

Studies show that a plethora of factors influence the uptake of CCS [22,23], as well as the lack of use and inadequate use of CCS [24]. Among these, social determinants of health play a central role. Women with higher education and employment status tend to benefit more from state-sponsored preventive healthcare opportunities, highlighting the importance of health literacy in public health initiatives. Other factors such as age, marital status [25–27], significantly influence the attendance of CCS programmes. Health-related behaviours may influence participation in CCS and may also be linked to underlying CC risk [28]. For instance, some evidence suggests that women with multiple sexual partners may be more likely to undergo CCS [22], while findings in other behaviours, such as condom use, are less consistent [29]. A meta-analysis has demonstrated that a higher number of lifetime sexual partners is independently associated with an increased risk of CC, independent of HPV status [30]. These findings highlight that behavioural risk factors and screening participation do not necessarily align, underscoring the importance of ensuring that screening programmes effectively reach women at increased risk, underscoring the importance of reaching high-risk groups.

In this context, the aim of our study was to evaluate compliance with the national CCS programme and to explore its associations with a range of factors, including socioeconomic and demographic characteristics, health status and behaviours, health-related knowledge, and access to healthcare services. We hypothesised that CCS compliance is significantly associated with socioeconomic and demographic characteristics, health status and behaviours, and knowledge related to CC prevention.

## Materials and methods

### Study design

A cross-sectional study was conducted between 1^st^ of March 2021 and 28^th^ of March 2022.

### Setting and study population

During the study period CCS in Latvia was performed at three-year intervals across the target population (women aged 25–70 years). Eligible women were invited to participate through invitation letters issued by the National Health Service. Screening was based on cytological testing. Women with abnormal cytological findings were referred for further diagnostic evaluation, including colposcopy, in accordance with the national CCS algorithm. Women referred for colposcopy were managed within the diagnostic and follow-up pathway of the CCS programme rather than separate CCS guidelines [6].

The present analysis is based on questionnaire data collected as part of a larger study assessing the prevalence of high-risk HPV and the feasibility of self-sampling for CCS, which has been described previously by Berza et al. [31].

The study population consisted of women aged 25–70 years residing in Latvia, recruited into two groups:

1. Women in the general population, with convenience sampling conducted through ten general practitioner (GP) practices selected to ensure coverage of all five Latvian regions, with two practices per region. Eligible women who attended these practices were consecutively invited to participate during routine visits until the target sample size was reached. Participating GPs were recruited during CC prevention conferences in 2020 and based on prior professional collaboration.
2. Women referred for colposcopy after abnormal cytological results were recruited at the Riga East University Hospital Colposcopy Unit, where all eligible women presenting during the study period were consecutively invited to participate at the time of their visit.

Eligibility criteria included women aged 25–70 years residning in Latvia, with sufficient proficiency in Latvian and Russian to complete the questionnaire. Women were excluded if they had a history of treatment for cervical precancerous lesions (e.g., excision or ablative procedures), as they were outside the target age range, or declined participation.

The proportion of women who declined participation was 11.1% in the general population group and 4.0% in the colposcopy group. The most reported reasons for non-participation included reluctance to perform self-sampling (47.1%), lack of time (20.6%), and the perception the recent gynaecological examination made participation unnecessary (14.7%).

This dual-population recruitment strategy allowed the inclusion of women in both the general population and women at elevated risk due to abnormal CCS results, thereby ensuring a broad and relevant basis for assessing CC prevention strategies and comparison of screening behaviours and awareness across different risk groups.

Participants recruited at a healthcare facility (such as a GP practice or the colposcopy clinic) provided written informed consent and completed a structured, self-administered paper questionnaire, gathering demographic, socioeconomic, health status and health behaviour data. The questionnaire was administered in Latvian and Russian to ensure accessibility.

Prior to data collection, the questionnaire was pretested in a clinical setting by trained gynaecology residents among women attending the colposcopy clinic to assess clarity and comprehensibility. Based on this process, minor refinements were made. The questionnaires were pseudonymized to ensure the confidentiality of the participants.

### Sample size

We used data from a study specifically powered to assess the prevalence of high-risk human papillomavirus (HR-HPV) among women. The data set includes information on 1279 women aged 25–70 years. A more detailed description of the sample size calculation is reported elsewhere [31].

### Variables

Outcome variable: odds of belonging to each CCS frequency category relative to the optimal screening category.

To analyse CCS uptake and assess over and underscreening, participants were categorised into four distinct groups based on their self-reported frequency of CCS. This categorisation aligns with Latvia’s national CCS programme in place during the study period, which recommended a 3-year CCS interval [6]:

1. Optimal CCS (reference category): women who reported having a cytological smear once every 3 years. This group represents adherence to the recommended CCS interval.
2. Over-screening: women who reported being screened once every 2 years or more frequently. This indicates a higher frequency than recommended by the national guidelines.
3. Under-screening: women who reported being screened once every 4 years or less frequently. This represents an inadequate or infrequent CCS pattern.
4. Unknown frequency: women who were unable to recall or did not know their CCS frequency were classified into a separate category of “do not recall.”

Independent variables. To identify factors associated with an inadequate frequency of participation in CCS, a set of independent variables was selected:

1. Sociodemographic variables (age, nationality, marital status, education, and financial situation of the household).
2. Health status (self-assessment of health and existence of chronic diseases).
3. Health behaviours (smoking status, binge drinking, condom use with a new sexual partner, and lifetime number of sexual partners).
4. CCS factors of health literacy. To quantify the knowledge and understanding of participants necessary for appropriate CCS decisions, factors related to CCS health literacy were assessed. These included awareness of the primary purpose of cytological screening (identifying cervical changes), general awareness of the HPV vaccine and the policies of the national CCS programme (invitation and frequency of performance every 3 years in Latvia). The last two items captured distinct aspects of CCS-related knowledge: awareness of the invitation interval reflects knowledge of the organised screening programme and its communication to the population, whereas awareness of the recommended screening frequency reflects understanding of the clinical screening interval itself. Potential multicollinearity between these variables was assessed prior to the model inclusion. The two variables were moderately correlated (Spearman’s ρ = 0.530, p < 0.001); however, this association was not accompanied by substantial variance inflation (VIF = 1.391 and tolerance = 0.719 for both variables). Therefore, both variables were retained in the model because they capture conceptually distinct dimensions of screening awareness.

A full list of study variables, including question formulation, response options, and categorisation, is provided in S1 Table.

### Data processing and statistical methods

Participant characteristics across the four CCS frequency groups were summarized descriptively. Overall differences in distributions across the four groups were assessed using Pearson’s chi-square test or Fisher’s exact test, as appropriate. Unadjusted omnibus p-values are reported in Table 1 for descriptive purposes only and were not used for covariate selection or for drawing the main inferential conclusions. To aid interpretation of descriptive patterns in Table 1, exploratory post-hoc comparisons of response-category contrasts between CCS frequency groups were conducted where applicable and are reported in S2 Table. These post-hoc comparisons were interpreted descriptively and were not used for covariate selection or for drawing the main inferential conclusions.

**Table 1:** Characteristics of the study population by frequency groups of CCS based on self-reported questionnaires, Latvia /2022/.

Independent variable	Over-screening		Optimal screening		Under-screening		Frequency not recalled		p	Total	
	n	%	n	%	n	%	n	%		n	%
Overall sample, n (%)	571	44.6	303	23.7	104	8.1	301	23.5	-	1279	100.0
**Sociodemographic characteristics**											
**Nationality**											
Latvian	426	74.6	233	76.9	69	66.3	226	75.1	0.202	954	74.6
Other	145	25.4	70	23.1	35	33.7	75	24.9		325	25.4
**Age**											
25-49	453	79.3	227	74.9	73	70.2	236	78.4	0.137	989	77.3
50+	118	20.7	76	25.1	31	29.8	65	21.6		290	22.7
**Marital status**											
Widowed/divorced	66	11.6	43	14.2	15	14.4	41	13.6	**0.002**	165	12.9
Married/cohabiting	428	75.0	216	71.3	73	70.2	187	62.1		904	70.7
Single	77	13.5	44	14.5	16	15.4	73	24.3		210	16.4
**Education**											
Primary/secondary/vocational	227	39.8	132	43.6	58	55.8	176	58.5	**<0.001**	593	46.4
University	344	60.2	171	56.4	46	44.2	125	41.5		686	53.6
**Financial situation of the household**											
Difficult	84	14.7	44	14.5	30	28.8	76	25.3	**<0.001**	234	18.3
Average	384	67.3	221	72.9	63	60.6	195	65.0		863	67.5
Good	103	18.0	38	12.5	11	10.6	29	9.7		181	14.2
**Health status and health behaviours**											
**Self-assessed health**											
Poor/average	209	36.6	116	38.3	43	41.3	140	46.5	**0.037**	508	39.7
Good	362	63.4	187	61.7	61	58.7	161	53.5		771	60.3
**Chronic diseases**											
No	380	66.5	184	60.7	72	69.2	210	69.8	0.101	846	66.1
Yes	191	33.5	119	39.3	32	30.8	91	30.2		433	33.9
**Smoking**											
Current	134	23.5	72	23.8	35	33.7	92	30.6	**0.022**	333	26.0
Former smoker	146	25.6	58	19.1	21	20.2	70	23.3		295	23.1
Never smoked	291	51.0	173	57.1	48	46.2	139	46.2		651	50.9
**Binge drinking**											
Once per week or more often	39	7.2	17	5.9	6	6.2	20	7.2	0.883	82	6.8
Once per month or less	506	92.8	273	94.1	91	93.8	256	92.8		1126	93.2
Using a condom with a new sexual partner within the last 6 months*											
No new partner	433	75.8	225	74.3	81	77.9	226	75.1	0.987	965	75.4
Never/rarely/occasionally	110	19.3	62	20.5	19	18.3	58	19.3		249	19.5
Always/almost always	28	4.9	16	5.3	4	3.8	17	5.6		65	5.1
**Number of sexual partners in a lifetime**											
4+	325	60.2	146	50.7	57	57.6	138	50.2	**0.014**	666	55.4
2-3	153	28.3	92	31.9	26	26.3	82	29.8		353	29.4
0-1	62	11.5	50	17.4	16	16.2	55	20.0		183	15.2
**CCS health literacy factors**											
**Awareness that the cytological screening test identifies cervical cell changes leading to cancer**											
No/don’t know	200	35.0	107	35.3	43	41.3	182	60.5	**<0.001**	532	41.6
Yes	371	65.0	196	64.7	61	58.7	119	39.5		747	58.4
**Awareness that women are invited to CCS every 3 years**											
No/don’t know	86	15.1	24	7.9	27	26.0	99	32.9	**<0.001**	236	18.5
Yes	485	84.9	279	92.1	77	74.0	202	67.1		1043	81.5
**Awareness that a cytological screening should be performed every 3 years**											
No	106	18.6	25	8.3	34	32.7	126	41.9	**<0.001**	291	22.8
Yes	465	81.4	278	91.7	70	67.3	175	58.1		988	77.2
**Aware of HPV vaccination as available preventive service**											
No	131	22.9	76	25.1	42	20.4	133	44.2	**<0.001**	382	29.9
Yes	440	77.1	227	74.9	62	59.6	168	55.8		897	70.1
**Venue of recruitment**											
Colposcopy group	250	43.8	93	30.7	39	37.5	108	35.9	**0.001**	490	38.3
General population group	321	56.2	210	69.3	65	62.5	193	64.1		789	61.7

*Notes for* Table 1*: n, number; p, p-value.*

*The p values are from Pearson’s chi-square test; Fisher’s exact test was used where expected cell counts were <5.*

*Statistically significant exploratory post-hoc response-category contrasts between CCS frequency groups are provided in* *S2 Table*.

*Percentages are based on the total study population; participants reporting no new sexual partners are included in the denominator.

To identify factors associated with CCS participation categories, bivariate association analyses and multivariable multinomial logistic regression models were fitted, using the optimal CCS category as the reference group. Multivariable models were adjusted for key sociodemographic characteristics: age, nationality, marital status, educational level, and household financial situation. In addition, recruitment setting (general population vs colposcopy referral) was included as a covariate to account for differences between subpopulations.

Given the number of statistical tests performed across multiple exposure variables and outcome categories, the Benjamini–Hochberg procedure [32] was applied to control the false discovery rate (FDR) (Table 2 and Table 3). Unadjusted p-values from the regression models are reported, and statistical significance was interpreted based on the BH-adjusted threshold (FDR = 0.05).

**Table 2:** Factors associated with CCS frequency (unadjusted analysis), Latvia /2022/ (reference category: optimal screening).

Independent variable	Overscreening			Underscreening			Frequency not recalled		
	OR	CI 95%	p	OR	CI 95%	p	OR	CI 95%	p
**Sociodemographic characteristics**									
**Nationality**									
Other	1.13	0.82–1.57	0.454	1.69	1.04–2.75	0.035	1.11	0.76–1.61	0.602
Latvian	1 (ref)	–	–	1 (ref)	–	–	1 (ref)	–	–
**Age**									
25-49	1.29	0.93–1.79	0.135	0.79	0.48–1.29	0.346	1.22	0.83–1.77	0.311
50+	1 (ref)	–	–	1 (ref)	–	–	1 (ref)	–	–
**Marital status**									
Widowed/divorced	0.88	0.51–1.50	0.630	0.96	0.42–2.18	0.921	0.58	0.33–1.02	0.056
Married/cohabiting	1.13	0.76–1.70	0.548	0.93	0.50–1.75	0.820	**0.52**	**0.34–0.80**	**0.003**
Single	1 (ref)	–	–	1 (ref)	–	–	1 (ref)	–	–
**Education**									
Primary/secondary/vocational	0.86	0.65–1.13	0.276	**1.63**	**1.04–2.56**	**0.032**	**1.82**	**1.32–2.52**	**<0.001**
University	1 (ref)	–	–	1 (ref)	–	–	1 (ref)	–	–
**Financial situation of the household**									
Difficult	0.70	0.42–1.19	0.187	2.36	1.04–5.33	0.040	**2.26**	**1.23–4.16**	**0.009**
Average	0.64	0.43–0.96	0.032	0.99	0.48–2.04	0.967	1.16	0.69–1.95	0.585
Good	1 (ref)	–	–	1 (ref)	–	–	1 (ref)	–	–
**Health status and health behaviours**									
**Self-assessed health**									
Poor/average	0.93	0.70–1.24	0.625	1.14	0.72–1.79	0.581	1.40	1.01–1.94	0.041
Good	1 (ref)	–	–	1 (ref)	–	–	1 (ref)	–	–
**Chronic diseases**									
No	1.29	0.96–1.72	0.087	1.46	0.90–2.34	0.122	**1.49**	**1.07–2.09**	**0.020**
Yes	1 (ref)	–	–	1 (ref)	–	–	1 (ref)	–	–
**Smoking**									
Current	1.11	0.79–1.56	0.563	1.75	1.05–2.93	0.033	**1.59**	**1.09–2.33**	**0.017**
Former smoker	1.50	1.05–2.14	0.027	1.31	0.72–2.36	0.379	1.50	0.99–2.27	0.054
Never smoked	1 (ref)	–	–	1 (ref)	–	–	1 (ref)	–	–
**Binge drinking**									
Once per week or more often	1.24	0.69–2.23	0.477	1.06	0.41–2.77	0.907	1.26	0.64–2.45	0.506
Once per month or less	1 (ref)	–	–	1 (ref)	–	–	1 (ref)	–	–
**Using a condom with a new sexual partner within the last 6 months**									
No new partner	1.10	0.58–2.08	0.769	1.44	0.47–4.43	0.525	0.95	0.47–1.92	0.876
Never/rarely/occasionally	1.01	0.51–2.02	0.969	1.23	0.37–4.11	0.742	0.88	0.41–1.90	0.746
Always/almost always	1 (ref)	–	–	1 (ref)	–	–	1 (ref)	–	–
**Number of sexual partners in a lifetime**									
4+	1.80	1.18–2.73	0.006	1.22	0.64–2.32	0.543	0.86	0.55–1.35	0.507
2-3	1.34	0.85–2.11	0.205	0.88	0.43–1.80	0.732	0.81	0.50–1.32	0.395
0-1	1 (ref)	–	–	1 (ref)	–	–	1 (ref)	–	–
**CCS health literacy factors**									
**Awareness that the cytological screening test identifies cervical cell changes leading to cancer**									
No/don’t know	0.99	0.74–1.32	0.933	1.29	0.82–2.04	0.272	**2.80**	**2.01–3.90**	**<0.001**
Yes	1 (ref)	–	–	1 (ref)	–	–	1 (ref)	–	–
**Awareness that women are invited to CCS every 3 years**									
No/don’t know	**2.06**	**1.28–3.32**	**0.003**	**4.08**	**2.23–7.47**	**<0.001**	**5.70**	**3.52–9.22**	**<0.001**
Yes	1 (ref)	–	–	1 (ref)	–	–	1	–	–
**Awareness that a cytological screening should be performed every 3 years**									
No	**2.54**	**1.60–4.02**	**<0.001**	**5.40**	**3.03–9.64**	**<0.001**	**8.01**	**5.01–12.80**	**<0.001**
Yes	1 (ref)	–	–	1 (ref)	–	–	1 (ref)	–	–
**Self-reported awareness of HPV vaccination**									
No	0.89	0.64–1.23	0.479	**2.02**	**1.27–3.24**	**0.003**	**2.37**	**1.67–3.34**	**<0.001**
Yes	1 (ref)	–	–	1 (ref)	–	–	1 (ref)	–	–
**Venue of recruitment**									
Colposcopy referrals	**1.76**	**1.31–2.36**	**<0.001**	1.36	0.85–2.16	0.202	1.26	0.90–1.77	0.176
General population sample	1 (ref)	–	–	1 (ref)	–	–	1 (ref)	–	–

*Notes for* Table 2*: OR, odds ratio; CI, Confidence Interval; p, p-value; ref, reference category.*

*P values are from Wald tests of multinominal logistic regression models.*

*Unadjusted p-values are reported; statistical significance was interpreted using the Benjamini–Hochberg false discovery rate (FDR) procedure (FDR = 0.05).*

*Bolded p-values indicate associations that remained statistically significant after FDR correction.*

**Table 3:** Factors associated with CCS frequency: Adjusted for socio-demographic variables, Latvia /2022/ (reference category: optimal screening).

Independent variable	Over-screening			Underscreening			Frequency not recalled		
	aOR	CI 95%	p	aOR	CI 95%	p	aOR	CI 95%	p
**Sociodemographic characteristics**									
**Nationality**									
Other	1.15	0.82–1.60	0.414	1.47	0.89–2.43	0.129	1.00	0.68–1.47	0.989
Latvian	1 (ref)	–	–	1 (ref)	–	–	1 (ref)	–	–
**Age**									
25-49	1.21	0.86–1.71	0.284	0.91	0.54–1.54	0.721	1.38	0.92–2.06	0.122
50+	1 (ref)	–	–	1 (ref)	–	–	1 (ref)	–	–
**Marital status**									
Widowed/divorced	0.95	0.55–1.66	0.866	0.77	0.33–1.82	0.557	0.57	0.32–1.04	0.067
Married/cohabiting	1.14	0.76–1.72	0.518	0.91	0.48–1.72	0.767	**0.54**	**0.35–0.83**	**0.005**
Single	1 (ref)	–	–	1 (ref)	–	–	1 (ref)	–	–
**Education**									
Primary/secondary/vocational	0.88	0.66–1.18	0.397	1.39	0.87–2.22	0.168	**1.70**	**1.22–2.38**	**0.002**
University	1 (ref)	–	–	1 (ref)	–	–	1 (ref)	–	–
**Financial situation of the household**									
Difficult	0.79	0.46–1.37	0.402	1.94	0.82–4.58	0.130	2.02	1.06–3.84	0.032
Average	0.68	0.45–1.03	0.070	0.94	0.45–1.96	0.858	1.11	0.65–1.88	0.712
Good	1 (ref)	–	–	1 (ref)	–	–	1 (ref)	–	–
**Health status and health behaviours**									
**Self-assessed health**									
Poor/average	1.01	0.74–1.38	0.928	0.85	0.52–1.40	0.522	1.20	0.84–1.71	0.307
Good	1 (ref)	–	–	1 (ref)	–	–	1 (ref)	–	–
**Chronic diseases**									
No	1.26	0.94–1.71	0.126	1.69	1.03–2.78	0.039	1.51	1.06–2.15	0.023
Yes	1 (ref)	–	–	1 (ref)	–	–	1 (ref)	–	–
**Smoking**									
Current	1.12	0.78–1.60	0.546	1.67	0.96–2.89	0.070	1.25	0.83–1.88	0.282
Former smoker	1.48	1.02–2.13	0.037	1.34	0.73–2.47	0.342	1.39	0.90–2.13	0.135
Never smoked	1 (ref)	–	–	1 (ref)	–	–	1 (ref)	–	–
**Binge drinking**									
Once per week or more often	1.24	0.68–2.24	0.483	1.01	0.38–2.65	0.991	1.11	0.56–2.19	0.760
Once per month or less	1 (ref)	–	–	1 (ref)	–	–	1 (ref)	–	–
**Using a condom with a new sexual partner within the last 6 months**									
No new partner	1.07	0.55–2.06	0.850	1.49	0.48–4.74	0.501	1.24	0.59–2.59	0.573
Never/rarely/occasionally	1.05	0.51–2.13	0.900	1.18	0.34–4.10	0.793	1.06	0.48–2.37	0.881
Always/almost always	1 (ref)	–	–	1 (ref)	–	–	1 (ref)	–	–
**Number of sexual partners in a lifetime**									
4+	**1.81**	**1.16–2.82**	**0.009**	1.29	0.65–2.55	0.471	0.62	0.38–1.01	0.056
2-3	1.34	0.85–2.13	0.210	0.86	0.41–1.78	0.680	0.66	0.40–1.09	0.104
0-1	1 (ref)	–	–	1 (ref)	–	–	1 (ref)	–	–
**CCS health literacy factors**									
**Awareness that the cytological screening test identifies cervical cell changes leading to cancer**									
No/don’t know	1.04	0.77–1.41	0.779	1.12	0.70–1.79	0.647	**2.57**	**1.83–3.62**	**<0.001**
Yes	1 (ref)	–	–	1 (ref)	–	–	1 (ref)	–	–
**Awareness that women are invited to CCS every 3 years**									
No/don’t know	**2.01**	**1.24–3.24**	**0.004**	**3.68**	**1.99–6.77**	**<0.001**	**5.54**	**3.40–9.03**	**<0.001**
Yes	1 (ref)	–	–	1 (ref)	–	–	1 (ref)	–	–
**Awareness that a cytological screening should be performed every 3 years**									
No	**2.52**	**1.58–4.01**	**<0.001**	**4.88**	**2.71–8.79**	**<0.001**	**7.30**	**4.54–11.73**	**<0.001**
Yes	1 (ref)	–	–	1 (ref)	–	–	1 (ref)	–	–
**Self-reported awareness of HPV vaccination**									
No	0.90	0.65–1.26	0.545	1.72	1.06–2.81	0.029	**2.00**	**1.40–2.87**	**<0.001**
Yes	1 (ref)	–	–	1 (ref)	–	–	1 (ref)	–	–
**Venue of recruitment**									
Colposcopy referrals	**1.77**	**1.30–2.40**	**<0.001**	1.30	0.80–2.12	0.287	1.09	0.76–1.55	0.649
General population sample	1 (ref)	–	–	1 (ref)	–	–	1 (ref)	–	–

*Notes for* Table 3*: aOR, adjusted odds ratio; CI, Confidence Interval, p, p-value; ref, reference category.*

*The p values are from Wald tests of multinominal logistic regression models.*

*Unadjusted p-values are reported; statistical significance was interpreted using the Benjamini-Hochberg false discovery rate (FD procedure (FDS = 0.05).*

*Bolded p-values indicate associations that remained statistically significant after FDR correction.*

Due to missing responses, the sum of categories for some variables may not equal the total study sample. Missing data was minimal for most variables; the highest omissions were observed for the lifetime number of sexual partners (6.0%) and binge drinking (5.6%). Analyses were conducted using available data for each variable (complete-case analysis), and no imputation was performed.

Data was analysed using IBM SPSS Statistics (version 29.0). Graphical visualisations were prepared in Microsoft Excel, and 95% confidence intervals (CIs) were additionally calculated using the OpenEpi calculator.

### Ethical considerations

The Riga Stradiņš University Ethics Committee approved the study (number of approval 6–1/07/ 33).

This study is reported according to the STROBE (Strengthening the Reporting of Observational Studies in Epidemiology) guidelines [33].

## Results

### Characteristics of the participants

A total of 1,279 women particpated in the study. Most of the participants in the study sample were Latvian (74.6%), aged 25–49 years (77.3%), married or cohabiting (70.7%), and held a university degree (53.6%). The majority assessed their financial situation as “average” (67.5%).

Regarding health status and behviours, more than half of the participants (60.3%) self-assessed their health as “good,” and 66.1% reported no chronic diseases. Slightly more than half of the participants (50.9%) were never-smokers, but binge drinking at least once a week was reported by 6.8% of study participants. Consistent condom use with a new partner was reported only by 5.1% of study participants, although this proportion includes women who reported no new sexual partners. In terms of lifetime sex partners, more than half (55.4%) reported four or more partners.

The awareness that the cytological screening can identify cervical cell changes leading to cancer was reported by slightly more than half of the respondents (58.4%), and awareness of the CCS invitations national regimen and the existence of the HPV vaccine was high (81.5% and 70.1% respectively). Among study participants, 38.3% represented colposcopy referral patients, and 61.7% – general population.

### CCS uptake

Based on self-reported screening frequency, 23.7% (95% CI 21.4–26.1, n = 303) of women adhered to the national CCS program recommendations, 44.6% (95% CI 41.9–47.4, n = 571) were categorized as overscreened, 8.1% (95% CI 6.8–9.8, n = 104) – underscreened and 23.5% (95% CI 21.3–25.9, n = 301) did not recall the frequency of participation in CCS. Participant characteristics, according to the division into four comparative groups depending on the frequency of participation in CCS, are reflected in Table 1.

### Factors associated with CCS uptake

In Table 1 sociodemographic, behavioural and knowledge-related characteristics among the CCS participation categories are described. Differences between groups were assessed using omnibus tests followed by post-hoc pairwise comparisons. Marital status, education level and financial status differed across CCS participation categories. Post-hoc pairwise comparisons showed that, compared with women who participated in CCS according to national guidelines (optimal screening group), those who did not recall their last CCS according to national guidelines were more likely to be single (p = 0.002), had lower educational attainment (p < 0.001), and were more likely to report poorer financial status (p < 0.001). Women in the optimal screening group were also more likely to have a university degree compared to both underscreened (p = 0.032) and “frequency not recalled” groups (p < 0.001).

Regarding CCS-related knowledge, women adhering to screening recommendations demonstrated greater awareness of the recommended screening interval compared to underscreened and “frequency not recalled” groups (both p < 0.001). They were also more likely to be aware of the role of cytological screening in detecting cervical cell changes (p < 0.001) and the HPV vaccination compared to underscreened (p < 0.001) and “frequency not recalled” groups (p < 0.001).

Additionally, women in the optimal screening group were more likely to be recruited from the general population compared to those who were overscreened (p < 0.001).

To further examine factors associated with CCS participation, regression analyses were performed (Table 2). The description is based on the CCS frequency groups.

Women who were unaware that screening invitations are issued every three years had higher odds of overscreening (OR = 2.06, 95% CI: 1.28–3.32), as did those unaware of the recommended screening interval (OR = 2.54, 95% CI: 1.60–4.02) compared to those who were aware. Additionally, women within the colposcopy referrals group had significantly higher odds of being overscreened (OR = 1.76 (95% CI 1.31–2.36)) compared to those from the general population. Underscreening was strongly associated with limited awareness of national CCS recommendations and preventive measures. Women unaware that CCS invitations are issued every three years (vs. aware) had significantly higher odds of underscreening (OR = 4.08 (95% CI 2.23–7.47)), as did those unaware of the recommended screening interval (vs. aware). (OR = 5.40 (95% CI 3.03–9.64)). Lack of awareness of the HPV vaccination was linked to underscreening (OR = 2.02 (95% CI 1.27–3.24)) compared to women who were aware of the HPV vaccination. Additionally, higher odds of underscreening were among women with primary, secondary, or vocational education (OR = 1.63 (95% CI 1.04–2.56)) compared to those with university education. Women who were unable to recall their last CCS visit formed a distinct group characterised by lower awareness and socioeconomic disadvantage. In unadjusted analysis (Table 2), lower education attainment (primary/secondary/vocational vs. university) (OR = 1.82, 95% CI 1.32–2.52) and financial hardship (difficult vs. good financial situation) (OR = 2.26, 95% CI 1.23–4.16) were associated with greater odds of uncertainty about CCS history. In contrast, being married was a protective factor for not being able to recall last CCS (OR = 0.52, 95% CI: 0.34–0.80) compared to being single. Women without chronic conditions compared to those with chronic diseases were more likely to fall into this group (OR = 1.49 (95% CI 1.07–2.09)), as were current smokers (vs. non-smokers) (OR = 1.59 (95% CI 1.09–2.33)). Lack of awareness emerged as a key factor in this group. Not knowing the purpose of cytological testing (OR = 2.80, 95% CI 2.01–3.90), the frequency of invitations (OR = 5.70, 95% CI: 3.52–9.22), or the recommended CCS interval (OR = 8.01, 95% CI 5.01–12.80) was strongly associated with not recalling last CCS visit in comparison to those being aware. Similarly, women unaware of the HPV vaccine had increased odds of recall uncertainty (OR = 2.37, 95% CI 1.67–3.34) in comparison to those aware.

After adjusting for sociodemographic factors in the multivariable analysis (Table 3), overscreening remained significantly associated with the number of lifetime sexual partners and limited awareness of national CCS guidelines. Women who reported four or more sexual partners in their lifetime (vs. 0–1) had 1.8 times higher odds (95% CI 1.16–2.82) of being in the overscreened group. Women unaware that CCS invitations are issued every three years had twice the odds of being overscreened (aOR = 2.01, 95% CI 1.24–3.24), and those unaware that cytological screening should occur every three years showed similarly increased odds (aOR = 2.52, 95% CI 1.58–4.01) in comparison to those aware. Similarly, women recruited from the colposcopy group were significantly more likely to be overscreened than those from the general population (OR = 1.77 (95% CI 1.30–2.40)).

Underscreening was significantly associated with lack of awareness about the CCS interval (OR = 3.68 (95% CI 1.99–6.77)) and lack of awareness that cytological screening should be done every three years (OR = 4.88 (95% CI 2.71–8.79)) in comparison to those aware.

All variables regarding CC prevention awareness were significantly associated with not being able to recall participation in CCS. Lack of awareness of the purpose of the cytological screening test and about the HPV vaccination was associated with approximately two times higher odds, namely aOR = 2.57 (95% CI 1.83–3.62) and aOR = 2.00 (95% CI 1.40–2.87) compared to those aware. Similarly, lack of awareness about the CCS invitation frequency (vs awareness) was strongly linked to being in the frequency-not-recalled group (aOR = 5.54 (95% CI 3.40–9.03)), as was lack of awareness about the recommended CCS interval (aOR = 7.30 (95% CI 4.54–11.73)) compared to those aware. Regarding sociodemographic factors, women with lower educational attainment (primary, secondary, or vocational) were more likely to not recall their CCS participation compared to university-educated women (aOR = 1.70 (95% CI 1.22–2.38)). Moreover, married or cohabiting women had significantly lower odds of not recalling last CCS compared to single women (aOR = 0.54 (95% CI 0.35–0.83)).

## Discussion

### Overall findings on CCS compliance

This study demonstrates substantial deviations from the CCS practices recommended by the guidelines in Latvia, where cytology-based screening at three-year intervals was predominantly used during the study period. Non-compliance with the 3-year guideline is widespread and takes three distinct forms. Overscreening was the most prominent deviation, reported by close to half of the women (44.6%). In contrast, a quarter of the women (23.7%) were adhering to the optimal CCS guideline. Underscreening affected 8.1% of the population. Equally striking is the large group of women whose CCS frequency was not recalled, accounting for a substantial proportion (23.5%) of the study population. This sizeable subgroup is characterised by uncertainty, suggesting limited engagement with CCS information, gaps in preventive health literacy, and weak integration of CCS into routine healthcare behaviours.

### Factors associated with overscreening

Overscreening was strongly associated with limited awareness of CC prevention. After adjustment for sociodemographic variables, women with a higher number of lifetime sexual partners, women with poor knowledge of national CCS guidelines, and those referred for colposcopy had significantly higher odds of undergoing CCS more frequently.

Women reporting four or more lifetime sexual partners had significantly higher odds of overscreening compared with those reporting zero or one partner (OR > 1.5 across models). Our finding aligns partially with observations from Denmark, where women with more sexual partners were significantly more likely to participate in CCS. It similarly demonstrates that women with higher sexual risk behaviours are more engaged with screening services [34]. Although findings are mixed, Gonzalez et al. [35] reported that women with fewer risky sexual behaviours were more likely to have undergone a CCS test in the previous three years than those with two or more.

CC prevention awareness-related variables emerged as some of the strongest indicators of overscreening. Women who were unaware that CCS invitations are issued at three-year intervals had twice the odds of overscreening (OR = 2.01; 95% CI: 1.24–3.24), and those who did not know that cytology is recommended every three years were 2.5 times more likely to be overscreened. These findings suggest that overscreening in Latvia is largely driven by low awareness of prevention guidelines. Castle et al. reported that both under- and overscreening were strongly tied to a lack of awareness or misunderstanding of national guideline intervals, emphasising that knowledge gaps can push women in both directions towards insufficient as well as unnecessary screening [36].

Women in the colposcopy referral group, representing those with previous abnormal findings or more intensive follow-up histories, also had significantly higher odds of overscreening compared to the general population. The overscreening in this group likely reflects both higher perceived risk and greater clinical contact, as previously reported in qualitative research from Estonia [37], where women with elevated risk expressed discomfort with less frequent screening and a strong preference for close monitoring.

Importantly, the high prevalence of overscreening observed in this study is likely multifactorial and cannot be explained by awareness alone. Patterns of healthcare utilisation could also affect overscreening. Women with more frequent contact with healthcare providers, particularly gynaecologists, may be more likely to undergo CCS at shorter intervals than recommended. Previous studies have shown that increased numbers of gynaecological visits and prior cervical testing, especially following abnormal results, are associated with higher odds of overscreening, likely reflecting opportunistic screening during routine care [11]. In addition, having a gynaecologist as the primary provider for CCS has been associated with a higher likelihood of screening overuse compared to primary care providers, suggesting an important role of provider-driven practices. This has led to recommendations for targeted interventions for gynaecologists and improved coordination between primary care physicians and gynaecologists to promote adherence to screening guidelines [38].

In Latvia, gynaecologists are directly accessible without referral, which may contribute to more frequent consultations and increase the likelihood that screening is performed during routine visits. Screening can also be accessed both within the publicly funded programme and through private healthcare services potentially driven by factors such as waiting times in the public sector, which may further facilitate more frequent testing.

Notably, in contrast to some European studies, the financial situation was not an independent predictor of overscreening. The association observed in crude models disappeared after adjusting for sociodemographic characteristics, suggesting that income-related differences in the frequency of CCS in Latvia may be mediated by health‐seeking behaviour, education, or access patterns rather than the socioeconomic status itself [39]. The finding contradicts other studies demonstrating that overscreening was more common among women with higher income, with significantly greater CCS uptake observed among those from wealthier households [40,41]. In contrast, lower income, financial instability, or declining income have consistently been associated with reduced participation in CCS [24,27,28,42–44].

Disparities in CCS uptake tend to be smaller in countries with organised programmes [45]. Latvia’s CCS programme is a state funded, organised initiative that includes gynaecological exams and cytology or HPV testing, performed by gynaecologists or general practitioners contracted with the NHS [6]. Alternatively, women can seek CCS through private gynaecologists who are often covered by private insurance, while CCS itself remains state funded. This dual-access model, combined with long waiting times in public care, has driven more affluent individuals toward private services [46]. A Serbian study [47] found that women with higher socioeconomic status were more likely to initiate CCS independently, whereas those with lower incomes relied more on organised or provider-recommended CCS. While similar disparities may exist in Latvia, current data do not differentiate between CCS conducted through public and private healthcare services.

### Factors associated with underscreening and unspecified frequency

Underscreened women were more likely to lack awareness of national guidelines regarding invitations to cervical cancer screening (CCS) and the recommended screening interval every three years, indicating that lower awareness may contribute to reduced participation in preventive activities. Women who were unable to recall their frequency of CCS formed an additional key subgroup. Approximately one-quarter of the participants were unable to recall whether they had attended CCS in the past three years. This uncertainty likely indicates lower participation in CCS, supported by the group’s limited awareness of national CCS recommendations and weaker knowledge of HPV vaccination, pointing to gaps in preventive health literacy. Evidence from Sweden and the Netherlands suggests that difficulty recalling the screening history is a marker of low engagement with organised screening programmes [25,29,48]. Similarly, a recent qualitative review by Wear and Shepherd [23] highlighted that misunderstandings about the purpose of screening, confusion between CC and other diseases, and inaccurate beliefs about the causes and symptoms of CC all hinder timely screening. However, the authors caution that lack of knowledge alone does not always predict non-attendance, indicating that multiple factors contribute to screening behaviour.

### The role of health literacy and knowledge gaps

Across all CCS patterns, health literacy and knowledge gaps emerged as central determinants of screening behaviour. Women who were over and underscreened were associated with lower awareness of CCS recommendations and limited awareness of the prevention services available within the country. Notably, nearly a quarter of women were unable to recall their history of CCS, indicating gaps in engagement and awareness. Although this study did not directly assess health literacy, the associations observed may reflect its role. Prior research shows a positive link between increased health literacy and enhanced participation in CCS, with expectations encouraging more women to use CCS services [49; 50]. However, the results of the studies are still contradictory. Although some concluded that low health literacy contributes to delayed or insufficient participation in CCS [22,51], others suggested that health literacy does not significantly influence cancer screening participation [52].

The proportion of participants classified as “other nationality” reflect the demographic composition of Latvia, which includes substantial ethnic diversity shaped by historical and geopolitical factors. Approximately 23% of the Latvian population identifies as Russian [53] and this proportion has remained relatively stable over time; therefore, the distribution observed in this study is consistent with the broader population structure.

Language may represent an important structural component of health literacy and access to organised screening programmes. Although Latvian is the sole official state language and health care services, including CCS, are delivered in Latvian [54], Russian is often used in daily communication by considerable share of the population. Similar considerations are observed in Finland, where immigrant background was operationalised using the variable “mother tongue other that Finnish” [55], reflecting the role of language as a potential barrier to engagement with routine CCS services. This consideration is particularly relevant in Latvia, where Latvian is mother tongue for 64% of the population [53]. Evidence from Estonia, a country with similar demographic composition, indicates that awareness of CCS is higher among Estonians compared with Russian-speaking women [56]. Together, these findings suggest that language-related factors may contribute to disparities in screening knowledge and participation within national screening programmes.

Evidence from high-performing European screening programmes highlights the critical role of knowledge in participation. In Estonia, only 72% of women were aware of the national programme, with language-based disparities pointing to persistent knowledge gaps even within organised systems [56]. In Sweden, non-attendance was primarily driven by practical barriers and limited awareness of screening-related information, rather than emotional concerns, emphasising the importance of informational clarity in communication [57]. A 24-country European analysis further demonstrated significant educational inequalities in CCS, attributed in part to differences in health literacy and comprehension of screening recommendations [58].

Higher health literacy has been associated with greater acceptance of new screening technologies and may mitigate the negative impact of low socioeconomic status on health behaviours [41,59]. Importantly, health literacy goes beyond mere knowledge of facts; it encompasses the ability to apply information to decision-making and self-advocacy when receiving preventive care. Therefore, improving health literacy can empower individuals to actively engage in decisions regarding screening uptake [60].

To better understand these dynamics, future research should frame CCS participation within a multidimensional health literacy model, such as that proposed by Galvin [61]. The implementation of novel, population-specific approaches such as culturally adapted health education, digital health tools, and community-based outreach has the potential to enhance CCS participation and coverage, ultimately contributing to more equitable and effective CC prevention.

### Implications for policy and public health interventions

The significant prevalence of overscreening and the large group of women unable to recall their frequency of CCS, along with the strong link to low health literacy, demand a fundamental policy shift in the Latvian CCS programme. First, health communication must be simplified and standardised for low-literacy audiences to reduce both overuse and participation gaps. More broadly, a consistently low awareness of key aspects of CC prevention indicates that current programme communication does not reach all women equally, revealing subgroups with lower health literacy who require more tailored and equity-oriented strategies. International evidence supports this approach; for example, co-designed and culturally tailored educational strategies are feasible, acceptable, and effective in improving engagement with CCS [62]. Evidence from European settings shows that interventions adapted to vulnerable groups, including co-designed educational materials and culturally tailored communication, can improve screening participation and support informed decision-making [63]. Second, inefficiencies at the system-level must be addressed. The current model allows unregulated re-screening outside the national programme, particularly in private care. Implementing a digital gatekeeping system in the public sector and better regulating interval adherence in private practice could help kerb unnecessary testing. The savings from reduced overuse could then be redirected to outreach efforts targeting underserved populations.

Taken together, our findings align with the WHO Europe Roadmap for Cervical Cancer Elimination (2022–2030), which prioritises improving public awareness through evidence-based communication strategies [64]. The observed coexistence of overscreening, underscreening, and uncertainty about screening history underscores the need for targeted, participatory approaches that address specific informational needs and structural barriers. These results suggest that generic population-wide messaging may not adequately reach all groups, highlighting the potential need of tailored interventions are essential to support informed participation across diverse population subgroups.

### Strengths and limitations

This study had several strengths. Data were collected from GP practices in all five Latvian regions, ensuring broad geographical coverage. The use of a structured questionnaire allowed the simultaneous assessment of sociodemographic, behavioural and knowledge-related determinants. Finally, by analysing overscreening alongside underscreening and recall uncertainty topics rarely addressed in Eastern Europe, the study offers novel insights relevant to ongoing transitions toward evidence-based HPV-based CCS in Europe.

This study had several limitations. First, the generalisability of the findings should be interpreted with caution. Although the study population aligned with the national age range of CCS (25–70 years), the use of a convenience sample introduces selection bias, limiting external validity. Second, the study included both women from the general population and women referred for colposcopy following abnormal CCS results. Although these groups are part of the same screening and diagnostic pathway, women in the colposcopy group may have higher underlying risk and more frequent contact with healthcare services, which may influence their screening behaviour. This could contribute to higher observed levels of overscreening in this group and potentially lead to an overestimation of overscreening prevalence in the combined sample. While recruitment setting was accounted for in the analysis, this should be considered when interpreting prevalence estimates of screening behaviours.

Third, the study relied on self-reported CCS histories and health information, which introduced risks of recall and social desirability bias. Misclassification of CCS frequency is possible, particularly if participants confuse cytology with HPV testing or misremembered the timing or setting of their last CCS. Fourth, the cross-sectional design prevents causal inference regarding the relationships between knowledge, health literacy, and screening behaviour. Non-response bias cannot be excluded, as women who chose not to participate may differ systematically in CCS engagement. Fifth, some CCS-related knowledge variables included in the analysis were conceptually related and may be correlated. Although no substantial multicollinearity was observed, these variables were retained to capture different dimensions of awareness. This should be considered when interpreting the findings. Finally, provider-level factors such as clinicians’ adherence to national guidelines or differences between public and private services were not captured, limiting our ability to contextualise overscreening patterns within the wider healthcare system.

## Conclusions

Limited awareness of the recommended 3-year CCS interval was strongly associated with both over and underscreening, and overscreening was also linked to reporting a higher number of lifetime sexual partners. Inability to recall the timing of the most recent CCS was consistently associated with lower CCS-related knowledge and awareness and was more common among women with lower educational attainment and those who were single. Overall, these findings highlight the critical role of health literacy in supporting informed and guideline-adherent participation. Implementing accessible and culturally tailored educational strategies can help reduce disparities and improve the equity and effectiveness of CC prevention efforts.

## Supporting information

S1 Table List of the variables used in the study. (DOCX)

S2 Table Statistically significant exploratory post-hoc response-category contrasts between CCS frequency groups for variables shown in Table 1. (DOCX)
