# Supplementary material for: Women’s compliance with cervical cancer screening recommendations and associated barriers to participation: A cross-sectional analysis
Source: PLoS One. 2026 Sep 10;21(9):e0357819. doi: 10.1371/journal.pone.0357819 (PMC13561318; doi:10.1371/journal.pone.0357819)
Supplement: Supplementary file 1 [file pone.0357819.s001.docx]

**Supplementary Table 1. List of the variables used in the study.**

| Variable | Question | Answers | Categorization |
| --- | --- | --- | --- |
| Outcome variables | | | |
| CCS frequency | How often have you had a cytological screening test done? | - More than once a year - Once a year - Once every two years - Once every three years - Once every four years - Once five or more years - Don’t know | - Optimal screening (Once every three years) - Underscreening (Once every four years and Once five or more years) - Overscreening (More than once a year and Once a year, and Once every two years) - Frequency not recalled (Don’t know) |
| Independent variables | | | |
| Nationality | What is your nationality? | - Latvian - Russian - Other |  |
| Age | In what year were you born? | Year _ _ _ _ |  |
| Marital status | What is your current marital status? | - Married/ registered partnership - Cohabiting - Divorced/Separated - Widow - Not married | - Widowed/divorced (Widow and Divorced/Separated) - Married/cohabiting (Married/ registered partnership and Cohabiting - Single (Not married) |
| Education | What is your education (the highest degree or level of school you have completed)? | - Elementary school (1–6 classes) - Middle school (7–9 classes) - High school (10–12 classes) - Vocational high school - Higher education | - Primary/secondary/vocational (Elementary school and Middle school, and High school, and Vocational high school) - Higher education |
| Financial situation of the household | How would you describe coping with your household’s income today? | - Living comfortably on present income - Coping on present income - Finding it difficult to cope on present income - Finding it very difficult to cope on present income | - Good (Living comfortably on present income) - Average (Coping on present income) - Difficult (Finding it difficult to cope on present income and Finding it very difficult to cope on present income) |
| Self-assessed health | In general, would you say your health is: | - Excellent - Very Good - Good - Fair - Poor | - Good (Excellent and Very good, and Good) - Poor/average (Fair and Poor) |
| Chronic diseases | Do you have long lasting health problems or a disease? | - Yes - No |  |
| Smoking | Do you smoke currently? | - Yes, every day - Yes, at least weekly - Yes, but less than weekly - No, have quit smoking - No, have never smoked | - Current (Yes, every day and Yes, at least weekly, and Yes, but less than weekly) - Former smoker (No, have quit smoking) - Never smoked (No, have never smoked) |
| Binge drinking | How often you drink a) three bottles (3x0,5 l) of beer or b) six glasses of (6x120 ml) wine or c) six shots of (6x40 ml) strong alcohol? | - Never - Less than once a month - Around once a month - Around once a week - Almost every day | - Once per week or more often (Around once a week and Almost every day) - Once per month or less (Never and Less than once a month, and Around once a month) |
| Using condom with a new sex partner within last 6 month | Did you use a condom (rubber) with a new partner during the past 6 months? *(a new partner is someone you never had sex with before this time period)* | - Haven’t had a new partner in the past 6 months - Never - Rarely - Sometimes - Almost always - Always | - No new partner (Haven’t had a new partner in the past 6 months) - Never/rarely/occasionally (Never and Rarely, and Sometimes) - Always/almost always (Almost always and Always) |
| Number of sex partners in a lifetime | Thinking back over your whole life, with how many partners have you had sexual intercourse? | _ _ number of sexual partners |  |
| Awareness that the cytological screening test identifies cervical cell changes leading to cancer | Does cytological screening test helps to reveal changes in cervical cells, that can lead without treatment to cervical cancer development? | - No - Yes - Don’t know | - No/don’t know - Yes |
| Awareness that women are invited to cervical screening every 3 years | Are women aged 30-55 invited to participate in cervical cancer screening taking cytological screening test every three years? | - No - Yes - Don’t know | - No/don’t know - Yes |
| Awareness that a cytological screening should be performed every 3 years | Do you know, that you need to have a cytological screening test every three years? | - No - Yes - Don’t know | - No/don’t know - Yes |
| Aware of HPV vaccination as available preventive service | Have you ever heard about a vaccination against human papillomavirus (HPV)? | - No - Yes |  |
| Venue of recruitment |  | - Women recruited in a colposcopy clinic - Women recruited among general practitioners | - Colposcopy group (Women recruited in a colposcopy clinic) - General population group (Women recruited among general practitioners) |
