# Supplementary material for: Women’s compliance with cervical cancer screening recommendations and associated barriers to participation: A cross-sectional analysis
Source: PLoS One. 2026 Sep 10;21(9):e0357819. doi: 10.1371/journal.pone.0357819 (PMC13561318; doi:10.1371/journal.pone.0357819)
Supplement: Supplementary file 2 [file pone.0357819.s002.docx]

**Supplementary Table 2. Statistically significant exploratory post-hoc response-category contrasts between CCS frequency groups for variables shown in Table 1.**

| Independent variable | Response-category contrast | Significant CCS group comparison | Post-hoc p-value |
| --- | --- | --- | --- |
| Sociodemographic characteristics | | | |
| Marital status | Married/cohabiting vs Single | Overscreening vs Frequency not recalled | <0.001 |
|  | Married/cohabiting vs Single | Optimal screening vs Frequency not recalled | <0.001 |
| Education | Primary/secondary/vocational vs University | Optimal screening vs Underscreening | 0.031 |
|  | Primary/secondary/vocational vs University | Overscreening vs Underscreening | 0.002 |
|  | Primary/secondary/vocational vs University | Overscreening vs Frequency not recalled | <0.001 |
|  | Primary/secondary/vocational vs University | Optimal screening vs Frequency not recalled | <0.001 |
| Financial situation of the household | Difficult vs Average | Optimal screening vs Underscreening | <0.001 |
|  | Average vs Good | Optimal screening vs Underscreening | <0.001 |
|  | Difficult vs Good | Optimal screening vs Underscreening | <0.001 |
|  | Difficult vs Average | Overscreening vs Underscreening | 0.002 |
|  | Difficult vs Good | Overscreening vs Underscreening | <0.001 |
|  | Difficult vs Average | Overscreening vs Frequency not recalled | <0.001 |
|  | Difficult vs Good | Overscreening vs Frequency not recalled | <0.001 |
|  | Average vs Good | Overscreening vs Frequency not recalled | <0.001 |
|  | Difficult vs Average | Optimal screening vs Frequency not recalled | <0.001 |
|  | Difficult vs Good | Optimal screening vs Frequency not recalled | 0.008 |
| Health status and health behaviours | | | |
| Self-assessed health | Poor/average vs Good | Overscreening vs Frequency not recalled | 0.005 |
|  | Poor/average vs Good | Optimal screening vs Frequency not recalled | 0.041 |
| Smoking | Current vs Never smoked | Optimal screening vs Frequency not recalled | 0.017 |
| Number of sexual partners in a lifetime | 4+ vs 0-1 | Overscreening vs Optimal screening | <0.001 |
|  | 4+ vs 2-3 | Overscreening vs Frequency not recalled | <0.001 |
|  | 2-3 vs 0-1 | Overscreening vs Frequency not recalled | 0.021 |
|  | 4+ vs 0-1 | Overscreening vs Frequency not recalled | <0.001 |
| CCS health literacy factors | | | |
| Awareness that the cytological screening test identifies cervical cell changes leading to cancer | No/don't know vs Yes | Underscreening vs Frequency not recalled | <0.001 |
|  | No/don't know vs Yes | Overscreening vs Frequency not recalled | <0.001 |
|  | No/don't know vs Yes | Optimal screening vs Frequency not recalled | <0.001 |
| Awareness that women are invited to CCS every 3 years | No/don't know vs Yes | Overscreening vs Optimal screening | 0.002 |
|  | No/don't know vs Yes | Optimal screening vs Underscreening | <0.001 |
|  | No/don't know vs Yes | Overscreening vs Underscreening | 0.006 |
|  | No/don't know vs Yes | Overscreening vs Frequency not recalled | <0.001 |
|  | No/don't know vs Yes | Optimal screening vs Frequency not recalled | <0.001 |
| Awareness that a cytological screening should be performed every 3 years | No vs Yes | Overscreening vs Optimal screening | <0.001 |
|  | No vs Yes | Optimal screening vs Underscreening | <0.001 |
|  | No vs Yes | Overscreening vs Underscreening | <0.001 |
|  | No vs Yes | Overscreening vs Frequency not recalled | <0.001 |
|  | No vs Yes | Optimal screening vs Frequency not recalled | <0.001 |
| Aware of HPV vaccination as available preventive service | No vs Yes | Optimal screening vs Underscreening | 0.003 |
|  | No vs Yes | Overscreening vs Underscreening | <0.001 |
|  | No vs Yes | Overscreening vs Frequency not recalled | <0.001 |
|  | No vs Yes | Optimal screening vs Frequency not recalled | <0.001 |
| Venue of recruitment | | | |
| Venue of recruitment | Colposcopy group vs General population group | Overscreening vs Optimal screening | <0.001 |
|  | Colposcopy group vs General population group | Overscreening vs Frequency not recalled | 0.024 |

Note. CCS, cervical cancer screening. This table identifies statistically significant exploratory post-hoc response-category contrasts between CCS frequency groups for variables shown in Table 1. Percentages for each response category and CCS frequency group are reported in Table 1 and are not repeated here. Only statistically significant post-hoc comparisons are displayed. Exact p-values are reported for p ≥ 0.001; p-values <0.001 are reported as p <0.001.
